# Bioinformatics Analysis Reveals Genes Involved in the Pathogenesis of Ameloblastoma and Keratocystic Odontogenic Tumor

**Published:** 2016-12-06

**Authors:** Eliane Macedo Sobrinho Santos, Hércules Otacílio Santos, Ivoneth dos Santos Dias, Sérgio Henrique Santos, Alfredo Maurício Batista de Paula, John David Feltenberger, André Luiz Sena Guimarães, Lucyana Conceição Farias

**Affiliations:** 1 *Department of Dentistry, Universidade Estadual de Montes Claros, Minas Gerais, Brazil.*; 2 *Instituto Federal do Norte de Minas Gerais-Campus Araçuaí, Minas Gerais, Brazil.*; 3 *Instituto Federal do Norte de Minas Gerais-Campus Salinas, Minas Gerais, Brazil.*; 4 *Department of Biology, Universidade Estadual de Montes Claros, Minas Gerais, Brazil.*; 5 *Department of Pharmacology, Universidade Federal de Minas Gerais, Brazil.*; 6 *Texas Tech University Health Science Center, Lubbock, TX, USA.*

**Keywords:** Ameloblastoma, keratocystic odontogenic tumor, cell proliferation, apoptosis, leader gene

## Abstract

Pathogenesis of odontogenic tumors is not well known. It is important to identify genetic deregulations and molecular alterations. This study aimed to investigate, through bioinformatic analysis, the possible genes involved in the pathogenesis of ameloblastoma (AM) and keratocystic odontogenic tumor (KCOT). Genes involved in the pathogenesis of AM and KCOT were identified in GeneCards. Gene list was expanded, and the gene interactions network was mapped using the STRING software. “Weighted number of links” (WNL) was calculated to identify “leader genes” (highest WNL). Genes were ranked by K-means method and Kruskal-Wallis test was used (P<0.001). Total interactions score (TIS) was also calculated using all interaction data generated by the STRING database, in order to achieve global connectivity for each gene. The topological and ontological analyses were performed using Cytoscape software and BinGO plugin. Literature review data was used to corroborate the bioinformatics data. *CDK1* was identified as leader gene for AM. In KCOT group, results show *PCNA* and *TP53*. Both tumors exhibit a power law behavior. Our topological analysis suggested leader genes possibly important in the pathogenesis of AM and KCOT, by clustering coefficient calculated for both odontogenic tumors (0.028 for AM, zero for KCOT). The results obtained in the scatter diagram suggest an important relationship of these genes with the molecular processes involved in AM and KCOT. Ontological analysis for both AM and KCOT demonstrated different mechanisms. Bioinformatics analyzes were confirmed through literature review. These results may suggest the involvement of promising genes for a better understanding of the pathogenesis of AM and KCOT.

Odontogenic tumors consist of a heterogeneous group of lesions that originate from the tissue that forms the teeth (1). These tumors affect individuals in different age groups, involving mandibular and maxillary region, with central or peripheral location. Some lesions are asymptomatic and are discovered by chance through routine radiographs. Additionally, odontogenic tumors could promote the local expansion or facial swelling (2, 3). Pathogenesis of odontogenic tumors is not well known. Several studies were performed to identify genetic deregulations and molecular alterations in an attempt to explain the mechanisms of oncogenesis, cytodifferentiation, and tumor progression (3, 4).

Ameloblastoma (AM) is a benign tumor originating in the odontogenic epithelium without ectomesenchyme, affecting the maxillo-mandibular complex (5). It is an asymptomatic lesion, and it presents locally invasive behavior, and higher recurrence rates (6). The differential diagnosis includes a variety of odontogenic cysts and tumors, particularly keratocyst odontogenic tumor and myxoma, non-odontogenic tumors and cysts, as central giant cell lesions and fibro-osseous lesions (7, 8).

The keratocystic odontogenic tumor (KCOT), according to the most recent classification of tumors of the head and neck of the World Health Organization (WHO), has been categorized as benign neoplasm derived from odontogenic epithelium. The great clinical relevance of KCOT is related to aggressive clinical behavior, high recu-rrence and proliferation rate (9, 10). However, there are still disagreements, questioning whether this odontogenic lesion indeed is a neoplasm or a cyst of odontogenic nature (11). Some studies have sought to understand these aspects through mole-cular investigations (11, 12).

Despite efforts focused on understanding the pathogenesis of odontogenic tumors, little is known about the real influence of molecular pathways and gene deregulations in these tumors. Silico approaches, such as bioinformatic analysis, have been performed to investigate signaling pathways, protein interactions, microRNA prediction models, and gene expression to obtain the best understanding of pathological mechanisms of diseases (13). The computational method is an important tool to understand molecular aspects of oral pathology and medicine (14-16).

This study aimed to investigate the differential involvement of protein-coding genes in the pathogenesis of AM and KCOT, through bioinformatics analysis.

## Materials and methods

**Bioinformatics and biological systems analysis**

Initially, key genes involved in the pathogenesis of AM and KCOT were identified by searching the GeneCards database (17). The gene nomenclature adopted was defined by Human Genome Organization (HUGO). The keywords, chosen according to Medical Subject Headings (MeSH), were “ameloblastoma and gene expression” and “keratocystic odontogenic tumor and gene expression”.

After this step, a list of potential “candidate genes” related to AM and KCOT was generated to each tumor. Then, this gene list was expanded using the web-available software STRING (version 9.1) (14), mapping the interaction network between these protein-coding genes. Direct and indirect gene interactions were considered with a high degree of confidence (above 0.9, range 0-0.99) (14)**. **With this process, new genes linked to AM and KCOT could be identified. For every gene interaction identified, we summed the interaction score of each gene, generating a combined association score. This score was adjusted, multiplying it by 1,000 (14), to obtain a single value called weighted number of links (WNL). The genes that showed the largest WNL values were named “leader genes” (14). Total interaction score (TIS) was also calculated using all interaction data generated by the STRING database to achieve global connectivity for each gene involved in the process (14). The value of WNL/TIS ratio represents the most influential genes in the network (specificity score). Genes with no link (orphan genes) were excluded from this analysis.

Genes were ranked according to this parameter in clusters, by the clustering method K-means. The number of clusters was calculated using the following equation: *Cluster number =TETO (LOG(CONT.NÚM(N);2);1)*. The number of clust-ers was obtained when mathematical convergence was achieved. To evaluate the differences among various classes based on WNL, Kruskal-Wallis test was used. Statistical significance was set at a p-value <0.001. Interacting genes were classified as up-regulated or down-regulated, as previously described (14), to each type of odontogenic tumor, AM or KCOT. Complementary analysis of biological systems was performed by topological and ontological analysis. The first was carried out with Cytoscape software (18), and ontological analysis was performed with BinGO plugin (14).

**Literature review**

A literature review was performed according to inclusion and exclusion criteria of the Preferred Reporting Items for Systematic Reviews and methodology Meta-Analyses (PRISMA). Literature review data were used to corroborate the bioinformatics data. The main question was "to verify genes that have been associated with AM or KCOT pathogenesis." The primary search was conducted in MEDLINE/PubMed database. The following keywords and their synonyms were used: "ameloblastoma and gene expression" and "kera-tocystic odontogenic tumor and gene expression." “Gene expression” corresponds to each of the genes obtained from GeneCard and leader genes.

The initial survey of the articles was conducted by three researchers (coauthors) according to the following inclusion criteria: articles published in English, with availability in its full version, whose study has been conducted with laboratory experiments and that addressed the topic of interest. After the initial selection of material, items with incompatible content with the object of the study were excluded. The final research material was made up of 66 selected papers for AM and 25 for KCOT. The synthesis of the collected data and evidence analysis are based on information regarding the characteristics, methods and study endpoints (title, aim, methodological design, sample, results, conclusion and level of evidence).

## Results

**Survey of genes associated with AM and KCOT pathogenesis and interaction network**

**Fig. 1.**
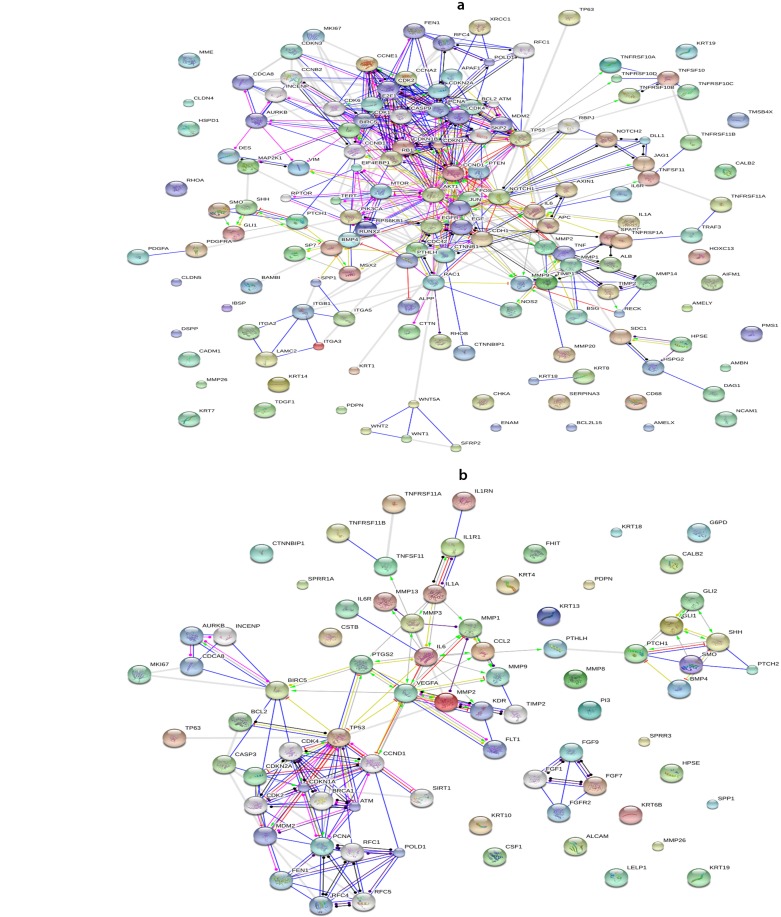
Gene interaction map and up- and down were regulated genes involved in ameloblastoma (a) and keratocyst odontogenic tumor (b). Data was derived from STRING (level of confidence > 0.9). Down-regulation is a red bar and up-regulation is a green arrow. Yellow circle represents that the directionality of the interaction is known, but it is not known whether it results from the interaction (e.g., if it is up- or down regulated). Black circle at both ends means some kind of interaction between the two proteins, but the directionality is not known. In deep blue: binding; in blue: phenotype; in indigo blue: catalysis; in violet: post-translation; in black: reaction; and in yellow: expression

The search through GeneCards and String database included 119 genes related to AM and 54 genes related to KCOT. Figures 1a and 1b show gene interaction maps and also increased and decreased gene expression in AM and KCOT, respectively. This figure also reveals the orphan genes. In the gene interaction network for AM, genes were the following: *AIFM1*, *AMBN*, *AMELX*, *AMELY*, *BCL2L15*, *CADM1*, *CALB2*, *CD68*, *CHKA*, *CLDN4*, *CLDN5*, *DSPP*, *ENAM*, *HOTAIR*, *HOXC13*, *HSPD1*, *IBSP*, *KRT14*, *KRT19*, *KRT7*, *MME*, *MMP26*, *NCAM1*, *PDPN*, *PMS1*, *RHOA*, *RUNX2*, *SERPINA3*, *TDGF1*, *TMSB4X*. furthermore, orphan genes in the network for KCOT were *ALCAM*, *CALB2*, *CSF1*, *CSTB*, *CTNNBIP1*, *FHIT*, *G6PD*, *HPSE*, *KRT10*, *KRT13*, *KRT18*, *KRT19*, *KRT4*, *KRT6B*, *LELP1*, *MIR15A*, *MIR16-1*, *MMP26*, *MMP8*, *PDPN*, *PECAM1*, *PI3*, *SPP1*, *SPRR1A*, *SPRR3*.

**Fig. 2:**
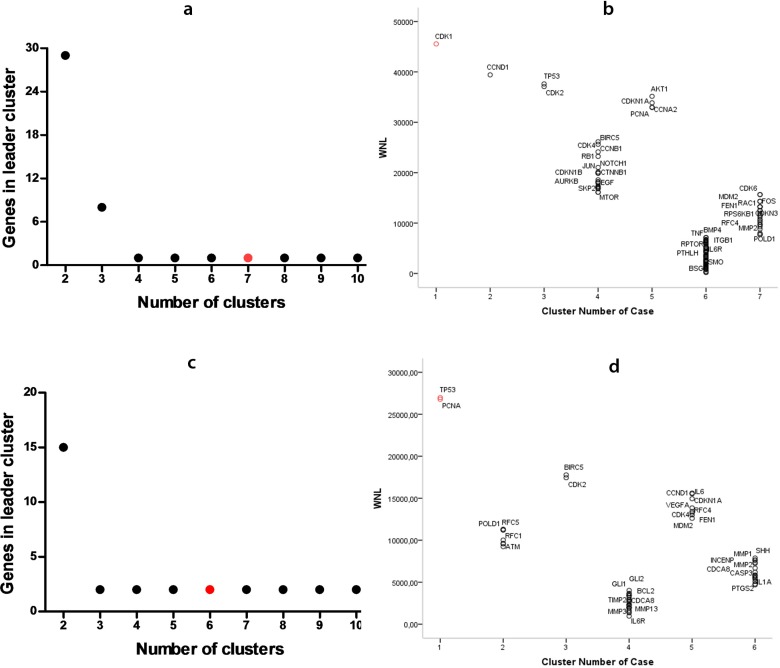
Data analysis of clustering for ameloblastoma (a and b) and keratocyst odontogenic tumor (c and d). Genes belonging to the leader cluster in different k-means clustering experiments with an increasing number of clusters. In red: number of clusters used (a and c); Number of cases in clusters with WNL for genes involved in the phenomenon. In red: gene leader cluster (b and d

Data analysis related to clustering and distribution of genes by cluster for AM and KOT diseases are represented in Figure 2. Figures 2a and 2c show the number of genes in the class of leader genes versus the increasing number of clusters; a preliminary k-means analysis revealed a cluster number equal to 7 for AM, and 6 for KCOT. Additionally, analysis points to the number of genes belonging to the leader cluster, being 1 for AM, and 2 for KCOT. Figures 2b and 2d show the number of genes in each class, and also demonstrate leader genes for each tumor. The WNL for each gene in the data sets are displayed in Figure 2. In AM, the highest WNL values were identified in genes *CDK1*, *CCND1*, *TP53*, *CDK2*, *AKT1*, *CCNA2*, *PCNA*, *CDKN1A*. Already, in KCOT, the most relevant WNL values were for *TP53*, *PCNA*, *BIRC5*, *CDK2*, *CCND1*, *CDKN1A*, *IL6*, *CDK4*, *VEGFA* genes. Clustering analysis of WNL identified only *CDK1* gene belonging to the largest cluster for AM. In the KCOT group, results show *PCNA* and *TP53* genes in the largest cluster.

**Fig. 3:**
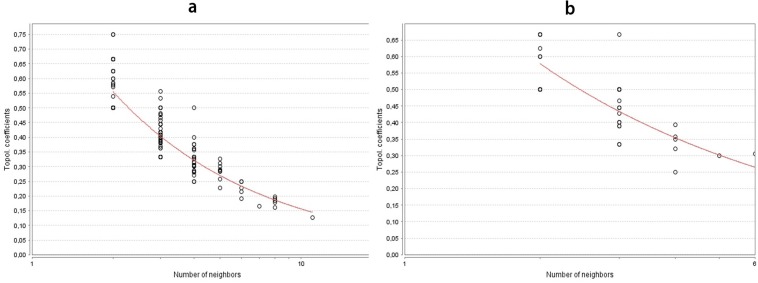
Power law behavior. a: ameloblastoma; b: keratocystic odontogenic tumor

Results were validated using the Kruskal-Wallis test, which revealed a statistically significant difference in WNL. In particular, the statistic analysis showed that leader genes had a signifi-cantly greater WNL than other classes of genes (P< 0.001). In this analysis, it was noted that both AM and KCOT tumors showed a power law behavior in agreement with the scale-free theory of network (In AM, correlation: 0.891; R^2^:0.855. In KCOT, corre-lation: 0.791; R^2^: 0.644)(Figure 3a and 3b). Power law distributions tend to differentiate nodes into specific points, meaning that some nodes have a tendency to have a low value, and consequently few number of connections, while other nodes, in turn, have a very high degree. In our case, we saw that few genes showed a large number of connections, whereas most of the genes showed few links. In this case, the high degree nodes are leader genes.

**Topological analysis**

**Fig. 4:**
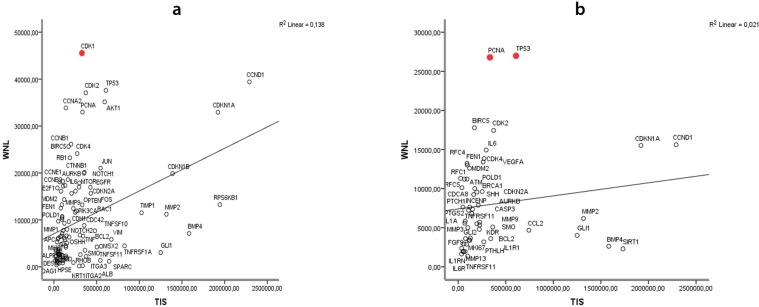
Scatter diagrams showing disease-related connectivities (WNL: weighted number of links) versus the global connectivities (TIS: total interactions score). a: ameloblastoma; b: keratocystic odontogenic tumor

A clustering coefficient was used to measure the degree of cohesion between the groups of genes. This numeric variable indicates the extent to which a gene is integrated into a given group. Clustering coefficient was close to zero (0.028) for AM, and zero for KCOT, demonstrating the importance of leader genes in connection between vertices and their neighbors (Table 1). When a gene appears above the regression line and very close to the Y axis, it means that it has a high specificity (WNL) and less global connectivity (TIS) suggesting that it is a leader gene. Figure 4a and 4b show the disease- related connectivities (WNL) versus the global connectivities (TIS). The WNL/TIS ratio indicated that the leader genes from AM (CDK) and KCOT (*PCNA* and *TP53*) were influential genes in the interaction networks.

**Table 1:** Global topological analysis for Ameloblastoma and Keratocystic odontogenic tumor network

**Parameter**	**Value**	**Parameter**	**Value**
**Ameloblastoma**			
Clustering coefficient	0.028	Number of nodes	150
Connected components	1	Network density	0.021
Network diameter	13	Network heterogeneity	0.510
Network radius	7	Isolated nodes	0
Network centralization	0.053	Number of self-loops	0
Shortest paths	13574 (100%)	Multi-edge node pairs	0
Characteristic path length	5.924	Analysis time (sec)	0.250
Avg. number of neighbors	3.187	-	-
**Keratocystic odontogenic tumor**			
Clustering coefficient	0.0	Number of nodes	34
Connected components	1	Network density	0.087
Network diameter	12	Network heterogeneity	0.355
Network radius	6	Isolated nodes	0
Network centralization	0.100	Number of self-loops	0
Shortest paths	1122 (100%)	Multi-edge node pairs	0
Characteristic path length	4.750	Analysis time (sec)	0.035
Avg. number of neighbors	2.882	-	-

**Ontological analysis**

**Fig. 5:**
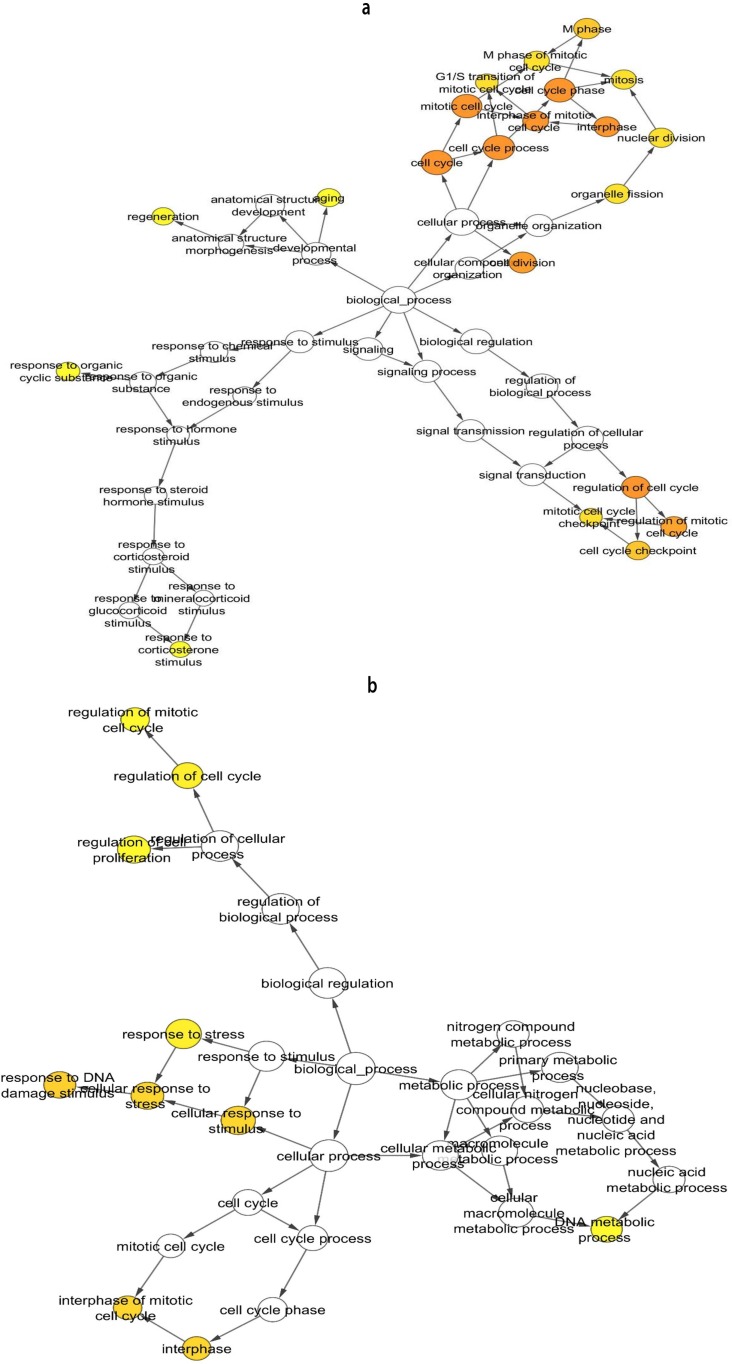
Ontology analysis of ameloblastoma (a) and Keratocystic odontogenic tumor (b) network. The most important pathways overrepresented in the graph versus whole set annotation, carried out with BinGO software (P < 0.01, Benjamini-Hochberg correction,hypergeometric clustering) are shown

The ontology for both AM and KCOT demonstrated different mechanisms associated to AM and KCOT (Figure 5a and 5b , respectively). In AM, aspects such as cell cycle process, regulation of cell cycle, regulation of mitotic cell cycle, interphase of mitotic cell cycle were relevant. In KCOT, regulation of DNA metabolic processes, mitotic cell cycle regulation, cell cycle regulation, as well as cellular response to a stimulus, cellular response to stress, and response to DNA damage were relevant. The mechanism of cell cycle regulation, for both AM and KCOT, was observed to be an outcome measure along with proliferation and anti-apoptotic mechanisms.

**Literature review**

Studies have demonstrated several genes related to AM and KCOT, but in literature, many controversies exist over whether it is involved in disease progression or, conversely, in tumor inhibition. So, we performed a short overview of literature regarding potentially involved genes and their expression in the pathogenesis of AM and KCOT. Expression levels (increase or decrease) of different genes were identified in this study, for both AM and KCOT, as summarized in Table 2.

**Table 2:** Gene expression in Ameloblastoma and Keratocystic odontogenic tumor according to the literature review.

**Gene ** **symbol**		**Level**	**In comparison of**	**Methodology**		**Ref.**
**AMELOBLASTOMA**						
CDK1		Expression of CDK1,-4, and - 6 was not changed, even with the induced overexpression of AMBN.	Primary dental epithelium bound to full-length AMBN	PCR and Western Blotting		(19)
CCND1		Increase	Keratocyts	Immunohistochemistry		(20)
TP53		Increase	Adenomatoid odontogenic tumor	*Immunohistochemistry*		(21)
CDK2		Overexpressed	Dentigerous cyst	Microarray analysis, RT-PCR, and immunohistochemistry		(22)
		No changed	No control group	Microarrays and immunohistochemistry		(23)
AKT1		Decrease	Under treatment of TNFα	Western blot/ Apoptosis assay/ DAPI staining		(24)
		Increase	Ameloblastoma tissues	**Immunohistochemistry/ Immunoblotting/Immunofluorescence/ELISA**		(21)
PCNA		Increased	Odontogenic keratocyst	Immunohistochemistry		(25)
CDK4		Increase	Rodent dental epithelial cell lines	Immunofluorescence/ immunohistochemistry		(26)
RB1		Increase	Cystic odontogenic tumor (CCOT)	PCR		(27)
JUN		Increase	Normal oral mucosa	*In situ* hybridization		(28)
CTNNB1		Increase	Oral basal cell carcinoma	Immunohistochemistry		(29)
NOTCH1		Decrease	Tooth germ	**Immunohistochemistry/ PCR Real Time**		(30)
		High	No comparison	Immunohistochemistry		(31)
MTOR		Increase	Dentigerous cysts (DCs), odontogenic keratocysts (OKCs)	Immunohistochemistry		(32)
EGFR		No expression	Normal oral mucosa	Immunohistochemistry		(33)
		Increase	Inflammatory cyst	Immunohistochemistry		(34)
E2F1		High	None	Immunohistochemistry		(35)
		Increase	Tooth germs	Immunohistochemistry		(36)
FOS		Increased	Other genes	Real-time PCR		(12)
MDM2		Increased	***Adenomatoid odontogenic tumor***	Immunohistochemistry		(37)
RAC1		No expression	None	Immunohistochemistry		(38)
PTEN		Decrease	Tooth germs	Immunohistochemistry		(39)
MMP9		Strongly expressed in mural, moderately in intraluminal, and weakly to absent in luminal variant	None	Immunohistochemistry		(40)
MMP2		Increased	Odontotheca tissues	Real-time PCR		(41)
MMP1		High	None	Immunohistochemistry		(42)
CDC42		No difference	Follicular and plexiform ameloblastomas	**Immunohistochemistry**		(38)
RUNX2		Low	None	PCR/ Western Blot/ Immunohistochemical		(43)
PTCH1		Same (High)	keratocystic odontogenic tumors	Immunohistochemistry.		(44)
BMP4		Expressed	None	Real- time PCR		(45)
MMP14		Higher in recurrent and solid/ multicystic ameloblastoma tissues than in primary and unicystic ameloblastoma tissues respectively	No control group	**PCR**		(41)
BCL2		High	P21	Immunohistochemistry		(46)
TIMP2		Low	Dentigerous cysts (DCs), radicular cysts (RCs), keratocystic odontogenic tumors (KOTs),	Immunohistochemistry		(47)
NOTCH2		No expression	Solid/multicystic (SA) and unicystic ameloblastomas (UA) recurrent ameloblastoma	Immunohistochemistry		(48)
TERT		Decreased	Oral mucosa	RT-PCR		(49)
CASP9		Same	Tooth germs	RT PCR		(50)
APC		Low	Tooth germs	Immunohistochemistry		(51)
VIM		Expressed	None	Immunohistochemistry		(52)
SHH		Low expression	Keratocystic Odontogenic tumor/ ameloblastoma	**Immunohistochemistry**		(44)
MKI67		Low	PCNA	Immunohistochemistry		(53)
TNFRSF1A		Increased	Tooth germ	RT PCR		(12)
MSX2		Decreased	P21 e p27	RT PCR Western blot		(53)
TNFSF11		Increased	Radicular cysts (RCs), dentigerous cysts (DCs)	Immunohistochemistry		(54)
APAF1		Same	Toothe germs	Immunohistochemical		(50)
SMO		Same (High)	keratocystic odontogenic tumors (KOT)	Immunohistochemistry		(44)
RECK		Low or no expression	keratocystic odontogenic tumor	Immunohistochemistry and RT-PCR		(55)
TNFRSF11B		Expressed	None	Immuhistochemistry, immunofluorescence and Western blot		(56)
		Lower	Tooth germs	RT-PCR and Immunohistochemistry		(57)
GLI1		High	Epithelial cells than in mesenchymal cells	RT-PCR and Immunohistochemistry		(58)
TRAF3		Low expression	Tooth germ	cDNA microarray and real-time RT-PCR		(12)
TNFRSF10B		Diffusely expressed	None	**Immunohistochemistry**		(59)
KRT18		Weakly and diffusely expressed	Tumor cells	Immunohistochemistry		(60)
KRT8		No expression.	None	**Immunohistochemistry**		(61)
TNFRSF10A		Diffusely expressed	None	Immunohistochemistry		(59)
TNFRSF11A		Expressed	Plexiform ameloblastomas than in follicular ameloblastomas	RT-PCR and Immunohistochemistry		(57)
NOS2		Increased	Tooth germs	Immunohistochemistry		(62)
WNT1		High	None	Immunohistochemistry		(63)
WNT5A		High	None	Immunohistochemistry		(64)
MAP2K1		Expressed	None	Immunohistochemistry		(65)
TNFRSF10C		Diffusely expressed	None	Immunohistochemistry and an in situ DNA nick-end labeling method		(59)
PDGFRA		Regularly expressed	None	Immunohistochemistry		(66)
SFRP2		Strongly	None	Immunohistochemistry and western blot		(67)
TP63		Higher	Plexiform ameloblastomas than in follicular ameloblastomas	Immunohistochemistry and RT-PCR		(68)
MMP20		No expression	None	Immunohistochemistry		(69)
PDGFA		High	Follicular ameloblastomas than in plexiform ameloblastomas	Immunohistochemistry		(70)
		Regularly expressed	None	Immunohistochemistry		(66)
SPARC		High	None	**Immunohistochemistry**		(71)
RHOB		High	Solid ameloblastoma.	Immunohistochemistry		(38)
WNT2		No expression or low	None	**Immunohistochemistry**		(63)
**KERATOCYSTIC ODONTOGENIC TUMOR**					
TP53	Low	Peripheral odontogenic keratocyst	Immunohistochemistry	(72)	
PCNA	Highest level was in the suprabasal layer of KCOT	Radicular cysts, dentigerous cysts, and calcifying cystic odontogenic tumors	Immunohistochemistry	(73)	
CCND1	Overexpression of downstream	Non-neoplastic oral mucosa	qPCR and immunohistochemistry	(44)	
IL6	Higher expression rates were associated with tumor size in ameloblastomas and with cyst wall thickness in keratocystic odontogenic tumors	Cases of ameloblastomas and cases of orthokeratinized odontogenic keratocysts	Immunohistochemistry	(74)	
VEGFA	High	Primary ameloblastoma, recurrent ameloblastoma, and malignant ameloblastoma	Immunohistochemical	(75)	
ALCAM	Deletion	No control group	Immunohistochemistry, Array-comparative Genomic Hybridization Labeling, qRT-PCR	(76)	
BCL2	Higher expression	KCOT and dental follicles	Immunohistochemistry,RNA isolation and quantitative reverse transcription (qRT-PCR) ,Western blotting	(77)	
CALB2	Expression less	KCOT and ameloblastoma	Immunohistochemistry	(78)	
CASP3	Express	KCOT, Ameloblastoma cisto radicular	immunohistochemical	(79)	
FGFR2	Express	OKC, dentigerous cyst, glandular odontogenic cyst, gingival cyst of the adult and the radicular cyst exhibited and normal dental follicles	Western blot analysis and immunohistochemistry	(80)	
FHIT	High	Dentigerous cysts (DC) and radicular cysts (RC)	Immunohistochemistry	(81)	
GLI1	Overexpression	None	Immunohistochemistry	(82)	
GLI2	Overexpression	None	Immunohistochemistry	(82)	
KRT6B	Over-expressed in 12q13	Tooth germs	Immunohistochemistry	(76)	
MKI67	Significantly higher	The cell proliferation and p53 protein expression (KCOT)	Immunohistochemistry	(83)	
MMP13	Positive labelling	Non-nevoid basal cell	Immunohistochemistry	(84)	
MMP9	Predominance	Follicular cyst	Immunohistochemistry	(85)	
PTCH1	Frequently detected genetic and/or epigenetic mechanisms of inactivation of the PTCH1 in KCOT	Genetic and/or epigenetic mechanisms of inactivation of the PTCH1 in KCOT	PTCH1 mRNA expression and methylation	(86)	
PTCH2	Germline mutations were detectable	Keratocystic odontogenic tumors	PCR-direct sequencing	(87)	
SHH	Expressed	None	Immunohistochemical	(88)	
SMO	No pathogenic mutation	NBCCS-associated KCOTs	Mutational analysis	(89)	
SPP1	Expressed	None	Immunohistochemistry	(90)	
TNFRSF11B	High	Solid ameloblastomas (SAs),	Immunohistochemistry using anti-RANKL and anti-OPG antibodies	(54)	
TNFSF11	High	Dentigerous cysts (DCs), solid ameloblastomas (SAs)	Immunohistochemistry using anti-RANKL and anti-OPG antibodies	(54)	
TP63	Higher	Epithelial lining of radicular cysts (RC), dentigerous cysts (DC)	Immunohistochemical	(75)	

## Discussion

AM and KCOT are odontogenic tumors strongly associated with cell proliferation and apoptosis inhibition (91,92). Literature reports have shown that an increase in the expression of caspase-3 and MMP-2 proteins are associated with growth and progression of KCOT and AM, indicating possible mechanisms involved in the recurrence KCOT and AM invasion. Moreover, epithelial lining of KCOTs showed a high turnover of cells suggesting that KCOT lesion can present neoplastic potential, as AM (79). Hence, studies have focused on molecular marker investigations for a better understanding of KCOT and AM pathogenesis.

In this sense, this study is the first to show, through bioinformatics analysis, interaction networks between protein- coding genes, leader genes and molecular pathways that can be related to the pathogenesis of AM and KCOT. Leader genes identified in the current study show distinct profiles between the odontogenic tumors; *CDK1* being the leader gene in AM, and *TP53* and *PCNA* as the leader genes in KCOT. All of these genes are somehow involved in apoptosis, cell cycle regulation, and cell proliferation.

The etiology and pathogenesis of AM are still not well understood. However, several factors such as *SHH*, *WNT*, *NOTCH*, *AKT* and *FGF* can be responsible for AM aggressiveness (93). In this context, it appears that both AM and KCOT are consequences of cell cycle deregulation, and/or apoptosis inhibition. The function of leader genes identified in this study coincides with the high proliferative activity of odontogenic epithelium in AM and KCOT. The literature reported a higher cell proliferation in KCOT than AM and similar apoptosis index between these tumors (94). These findings can support the classification of KCOT as an odontogenic tumor and can be related to its aggressive clinical behavior (94). Similarly, another study showed the aggressive nature of KCOT. KCOT and AM have been clearly demonstrated to have both intrinsic growth potential and aggressive invasive behavior (95).

Interestingly, no study was found in the literature aiming to evaluate specifically the role of *CDK1* gene, identified by our bioinformatics analysis as a leader gene in AM. Our literature search revealed a single study where *CDK1 *expression was reported in human ameloblastoma AM-1 cells (19). The expression of *CDK1*, *-4*, and *-6* was not changed in AM-1 cells, even with the induced overexpression of ameloblastin gene that may function as a tumor suppressor. Therefore, the *CDKs* remained expressed in the AM-1 cells (19). The CDK1-cyclin B complex is essential to initiate mitosis and can phosphorylate a wide range of proteins involved in regulatory and structural processes necessary for mitosis such as the nuclear envelope breakdown, condensation of chromatin, fragmentation of the golgi apparatus, and training of mitotic spindles (96).

Other studies have evaluated gene expression of other cyclin-dependent kinases and their receptors. Cyclin D1 was expressed in epithelial cells near the basement membrane in dental germs and AM. This fact seems to indicate that this protein is involved in cell proliferation in odontogenic epithelium and ameloblastic tumors (97, 98). The mRNA expression of cyclin E increased with AM recurrence and malignant transformation suggesting that genesis and invasion of AM are associated with the cell proliferation and differentiation, and are well-regulated by the increased expression of cyclin E and the lower expression of p21(*WAF1*) and p27(*KIP1*) (99).

Despite the scarcity of studies on *CDK1* in AM, this gene was appointed as a leader gene due to the increased number of interaction networks that it establishes with other genes, especially related to increased activity of cell proliferation, as demonstrated in the analysis of clustering and ontology. In addition to leader genes, the bioinformatic analysis shows several genes possibly related to this tumor, such as *TP53*, *AKT1*, *PCNA*, *PTCH*, *mTOR, MMPs*, *BCL2* as well as others. By bioinformatics data, our literature review indicated that hedgehog signaling genes such as *HH*, *PTCH1*, *SMO*, and *GLI* are involved in AM pathogenesis. A high expression of SHH, SMO and GLI protein was reported in AM (4). Hedgehog (HH) signaling is a conserved pathway that guides embryonic patter-ning through the temporal and spatial regulation of cellular proliferation and differentiation (100). The HH pathway contributes to iASPP (inhibitor of the apoptosis-stimulating protein of p53) function, through the activation of Cyclin B1 and by the E2F1-dependent regulation of *CDK1*. These mecha-nisms are involved in iASPP induction. Results showed that activation of HH signaling enhances proliferation in the presence of E2F1 and contri-butes to apoptosis in its absence or upon CDK1 inhibition (101). In this bioinformatics study, the *CDK1* gene was considered as the leader of the molecular pathway of AM pathogenesis, demons-trating the importance of genes of the HH pathway in the pathogenesis of AM through the leader gene.

*TP53* and *PCNA *were identified as KCOT leader genes. Immunohistochemical studies have examined KCOT by using various markers of proliferation and apoptosis (102, 103). Proliferative activity of the epithelial lining of KCOT has been the subject of various investigations examining p53 expression (102), proliferating cell nuclear antigen (PCNA) (103) and Ki-67 (104). These studies concluded that KCOT displays a greater expression of p53, PCNA, and Ki-67 as compared to other types of odontogenic cysts (105).

The first leader gene in KCOT was the *TP53*, which is consistent with the available scientific information about its role in tumor development (106). *TP53* is a tumor suppressor gene with effective action at the G1 phase of the cell cycle, which participates in growth arrest, initiates repair, or induces apoptosis (107). Immunodetection of this protein seems to influence the stabilization of the p53 product, an action which interferes with cell cycle regulation for proliferation (108), indicating an intrinsic growth potential of the KCOT epithelium (109). On the other hand, the literature review revealed weak expression of p53 in odontogenic lesions, such as KCOT and peripheral odontogenic keratocyst, in the same way as normal gingiva (9).

The second leader gene in KCOT was *PCNA*, encoding a nuclear protein linked to DNA replication and initiation of cell proliferation. Increased expression of PCNA may be stimulated with growth factors or as a result of DNA injury (110).

Molecular expression of p53 and PCNA in different odontogenic lesions revealed the highest level of both proteins in the suprabasal layer of KCOT compared with radicular cyst, dentigerous cyst, and Gorlin cyst, suggesting that proliferation and maturation patterns in KCOT differ from those found in the other lesions (9). Overexpression of *PCNA* in the suprabasal layer of the KCOT can clarify its neoplastic nature and a tendency toward tumor recurrence (73).

Similar to the AM, results revealed *PTCH* as a gene importantly involved in interaction network in KCOT. Previous studies showed evidence of mutations in the *PTCH* gene (89, 111), a tumor suppressor associated with nevoid basal cell carcinoma syndrome. Mutations in this gene could be responsible for migration and differentiation of abnormal cells, and also might interfere with apoptosis, which would lead to a deregulation of cell proliferation (112). Nonetheless, this gene was not designated as one of the leaders in our bioinformatics analysis, but it was highlighted by relevance described in the literature in both KCOT and AM (113, 114).

The analysis of interaction map allowed the identification of different groups of genes acting on cell cycle regulation in AM and KCOT. This search confirmed that each gene identified as a leader gene could supposedly play an important role in AM and KCOT. Nevertheless, literature survey also revealed that other genes might be potentially involved in the pathogenesis of AM and KCOT, as shown in Table 2.

In our bioinformatics analysis, the interaction network of leader genes with a significant number of genes of the apoptosis pathway suggests that cell cycle deregulation is an important molecular event in both AM and KCOT. Moreover, functional enrichments in our network obtained from STRING database showed that orphan genes not linked to leader genes are ontologically related to the cell adhesion molecules and structural molecule activity.

Bioinformatics conducts a theoretical analysis using public databases, gene database, and scientific publishing databases, to generate relevant information and new knowledge. These theoretical results are well-supported by findings in the literature on the contribution of genes to the pathogenesis of AM and KCOT. Thus, based on the bioinformatics results and literature survey, further laboratory experiments should be conducted to better explain the real importance of interaction networks between genes in the pathogenesis of odontogenic tumors.

These data obtained through bioinformatics analysis can contribute to an improved body of knowledge about genes and molecular mechanisms involved in AM and KCOT pathogenesis. It is noteworthy that a detailed analysis of gene interaction networks and molecular pathways can be of great value in identifying new targets for an understanding of diseases and also to point to possible therapeutic targets. In addition, bioinformatics analysis can be an important tool for designing a hypothesis before conducting functional studies.

In this study, some genes with an important potential role in the pathogenesis of AM and KCOT were identified. Even with the limitations of any theoretical study, these preliminary results may suggest the involvement of promising genes for a better understanding of these odontogenic tumors.
